# Aloe vera supplementation improves cardiovascular risk factors in hemodialysis patients: A randomized, double-blind, placebo-controlled trial

**DOI:** 10.22038/AJP.2023.23447

**Published:** 2024

**Authors:** Akram Kooshki, Mohammad Reza Memarzadeh, Mohammad Hassan Rakhshani, Roya Akbarzadeh, Tahereh Tofighiyan, Elaheh Foroumandi

**Affiliations:** 1 *Non-Communicable Diseases Research Center, Department of Nutrition & Biochemistry, School of Medicine, Sabzevar University of Medical Sciences, Sabzevar, Iran*; 2 *Barij Medicinal Plants Research Center, Kashan, Iran *; 3 *Department of Environmental Health, School of Health, Sabzevar University of Medical Sciences, Sabzevar, Iran*; 4 *Department of Anesthesiology, Faculty of Paramedical, Sabzevar University of Medical Sciences, Sabzevar, Iran*; 5 *Department of Nursing, School of Nursing and Midwifery, Sabzevar University of Medical Sciences, Sabzevar, Iran*

**Keywords:** Aloe vera, C-reactive protein Inflammation, Lipids, Hemodialysis

## Abstract

**Objective:**

This study assessed the effects of Aloe vera supplementation on serum inflammatory factors, blood sugar and lipid profiles in hemodialysis patients.

**Materials and Methods:**

Totally, 50 hemodialysis patients were allocated randomly to either Aloe vera or placebo groups. The Aloe vera group received 2 Aloe vera capsules daily for 8 weeks (500 mg/day). Serum C-reactive protein (hs- CRP), Fasting blood glucose (FBS), and lipid profiles levels were evaluated at the baseline and the end of the eighth week.

**Results:**

Aloe vera supplementation for 8 weeks was associated with a significant reduction of serum hs- CRP (p=0.004), total cholesterol (p=0.01), low density lipoprotein (LDL) (p=0.02) leves and increased high density lipoprotein (HDL) (p=0.002) concentration in the hemodialysis patients.

**Conclusion:**

Aloe vera supplementation is beneficial in improvement of cardiovascular risk factors in hemodialysis patients.

## Introduction

Chronic hemodialysis patients are at high risks of morbidity and mortality, and they experience a low quality of life (La Russa et al., 2019 ▶). Cardiovascular disease (CVD) is a major cause of death in chronic kidney patients (Jungers et al., 1999 ▶). Inflammation, lipid abnormalities, oxidative stress, and hypertension are the major risk factors for CVD in hemodialysis patients (Jungers et al., 1999 ▶). Inflammatory markers such as c-reactive proteins (CRP) and oxidative stress indices increase in chronic kidney disease (CKD) and hemodialysis patients (Yari et al., 2020 ▶). Further, lipid abnormalities such as high levels of serum triglyceride (TG), total cholesterol (TC), low-density lipoprotein cholesterol (LDL-C) and low levels of serum high-density lipoprotein cholesterol (HDL-C) are one of the major risk factors for CVDs in these patients (Jungers et al., 1999 ▶). HDL-C has multi-protective effects such as vaso-protective and anti-inflammatory actions, which can be impaired in CKD and particularly in hemodialysis patients (Kooshki et al., 2019 ▶).

Recently, most of the people have access to variety of traditional medicine and they have tendency to use medicinal plants (Foroumandi et al., 2023 ▶). Aloe vera, as a medicinal herb is used for many therapeutic purposes. It is from the Mediterranean region, Arabian Peninsula, India, China, and Eastern Africa (Florens et al., 2019 ▶). Aloe vera has a large amount of bioactive compounds such as flavonoids, terpenoids, lectins, fatty acids, anthraquinones, mono saccharides, fibers (pectins, hemicelluloses, and glucomanan), tannins, phytosterols (campesterol, and B-sitosterol), enzymes, salicylic acid, minerals (calcium, chromium, iron, copper, zinc, magnesium, manganese, potassium, phosphorus and sodium), and vitamins (A, B-carotene, C, E, B-complex) (Marzanna et al., 2019 ▶). There is evidence that Aloe vera has many medical properties such as lowering blood sugar by improving sensitivity to insulin in diabetic patients (Choudhary et al., 2014 ▶; Shahryari et al., 2021 ▶), reducing serum TC, triglycerides, and blood pressure and increasing the levels of HDL-C (Vinay et al., 2012 ▶). It inhibits cyclooxygenase pathway and reduce prostaglandin E_2 _(PGE_2_). Also, it contains bradykinin peptidase enzyme which helps breakdown the bradykinin (an inflammatory substance that causes pain) and decreases the inflammation (Gupta et al., 2017 ▶). Further, some recent studies have indicated that Aloe vera can significantly reduce oxidative stress by increasing concentration of glutathione peroxides, superoxide dismutase, phenolic antioxidant (Sahu et al., 2013 ▶; Keshavarzi et al., 2014 ▶), vitamins A, C, and E and carotenoids (Sahu et al., 2013 ▶; Marzanna et al., 2019 ▶).

Therefore, this study was the first clinical trial study that was done to demonstrate the effects of Aloe vera supplementation on cardiovascular risk factors in hemodialysis patients.

## Materials and Methods

**Participant**

This study was conducted on the patients who were recruited from the hemodialysis clinic of Vaseii Hospital of the University of Sabzevar, Iran. The study was approved by the Research and Ethical committee of Sabzevar University of Medical Sciences (No: IR.Medsab.Rec.1395.16). Further, the trial was registered in the Iranian website (www.irct.ir) for registration of clinical trials (IRCT code: IRCT20180122038472n3).

**Intervention**

The inclusion criteria were the patients who were dialyzed with polysulfone capillary dialyzers 3 times a week for 4 hr per session. The subjects who had history of any inflammatory or infectious disorders were excluded. Further, the people who had received Aloe vera, omega-3 fatty acid, L-carnitine, vitamin E and/or C supplements, or steroidal and/or non -steroidal anti-inflammatory drugs were excluded. The hemodialysis procedure and type of the dialyzer were not altered during the study. After explaining the process and objectives of the study to each patient, informed consents were signed by them. Subjects in the Aloe vera group received 2 Aloe vera capsules (500 mg/day) daily for 8 weeks, while the placebo group received 2 placebo capsules daily. Aloe vera and placebo capsules were prepared by Barij Essence Kashan Company. All the capsules were at the medium size and white color and had a similar appearance. All the researchers and patients were blinded to the type of supplements that were received by the participants.

All participants were advised not to change their lifestyle, diet and physical activity during the study. International physical activity questionnaire (IPAQ) and a 24-hour food recall questionnaire were filled to ensure that these items did not change. The compliance of the patients was assessed according to the number of returned capsules that was more than 90%. No side effects were reported with this supplement.

**Blood samples and analysis**

Totally, 5 ml blood sample was collected from each patient at the baseline and the end of the study, after a 12 to 14 hr fasting. Blood samples were centrifuged at room temperature (20–25°C) at 2,000 rpm for 10 min. The obtained serum was frozen and kept at –20°C until analyses.

The serum hs-CRP concentration was determined using enzyme-linked immune sorbent assay (ELISA) kits (Monobind, Inc., Lake Forest, Calif., USA). Serum concentrations of FBS, TG, TC, LDL –C, and HDL-C were measured using commercial kits (Pars Azemoon Co., Tehran, Iran), by Hitachi 717 auto-analyzer (Boehringer Mannheim Diagnostics, Mannheim, Germany). 

**Sample size**

The sample size of the study was determined according to formula “n= (Z 1- α/2 + Z 1- β) 2 (σ12 + σ22)/ (μ12 - μ 22)”, considering α=0.05 and power test of 0.9. Considering Nasab et al, study 46 hemodialysis patients were included in the study using convenience sampling method (Nasab GA et al., 2012 ▶).

**Statistical analysis**

Statistical analysis of the study was performed using SPSS 20 (SPSS, Inc., Chicago, Ill., USA). All the quantitative parameters were normally distributed according to the Shapiro-Wilk test. The differences between investigated risk factors at the baseline and end of the study were analyzed using Paired Sample t-test. Analyses covariance (ANCOVA) was also performed to compare investigated parameters between the groups. The models were further adjusted for a priori selected set of variables including age, sex, body mass index (BMI), and duration of the hemodialysis. A significance level of 0.05 was considered for all analyses.

## Results

**Figure 1:**
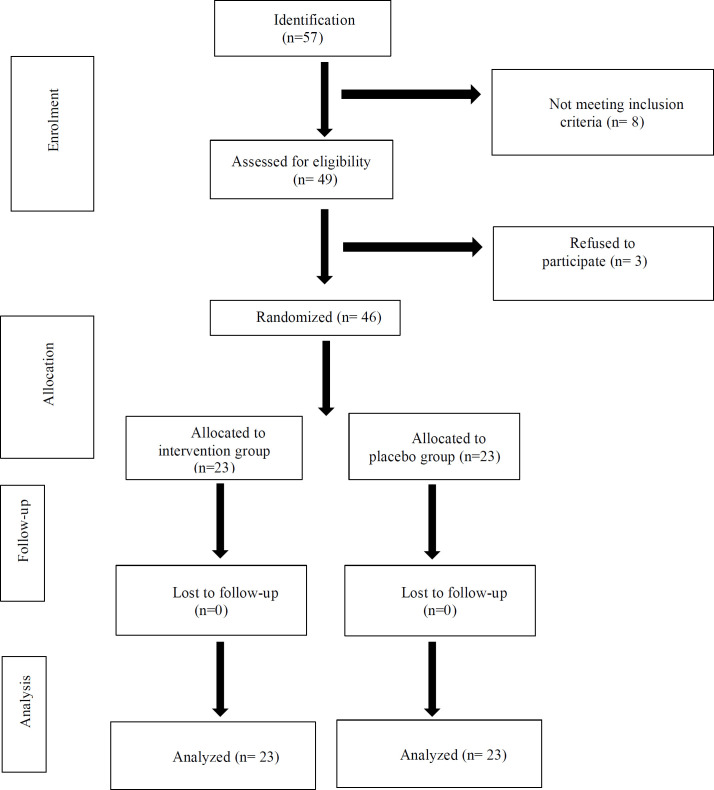
Flowchart of the study

Totally, 46 patients were participated in the study (Figure 1), as 23 (46%) of them were female and the age range of the patients was 40-75 years. The mean (±standard deviation (SD)) age of the patients was 58.2 (±13.5) and 57.05 (±17.7) years in the Aloe vera and placebo groups, respectively. The duration of hemodialysis was 3.3 (±2.7) and 4.5 (±2.9) years in Aloe vera and placebo groups, respectively. The age, gender, duration of disease, and BMI were not significantly differ tent between the groups (Table1).

Fasting blood glucose, lipid profiles, and hs-CRP levels at the baseline of the study and after 8 weeks are presented in Table 2. As seen in Table 2, serum TC (p=0.01), LDL-C (p=0.02), and hs-CRP (p=0.004) status decreased in the Aloe vera group compared to the baseline, while there was not any significant change in the placebo group. Further, the concentration of serum HDL-C in the Aloe vera group was increased compared to the baseline (p=0.002). 

As seen in Table 3, Aloe vera supplementation was effective in decreasing hs-CRP levels by 10.72 mg/L compared to the placebo group after adjusting the analysis for confounding factors including sex, age, duration of hemodialysis, BMI, and smoking.

**Table 1:** Baseline characteristics and anthropometric data of dialysis patients in the Aloe vera and placebo groups

Placebo (N=23)	Aloe vera (N=23)	characteristics
57.05±17.70	58.26±13.55	Age (years)
4.58±2.95	3.31±2.71	Duration of diabetes (years)
		sex
10(52.6%)	17)54.8% (	Men, n
9(47.4%)	14(45.2%)	Women, n
9(47.4%)	12(38.7%)	Smoker, n
24.03±6.01	25.48±3.73	BMI (kg/m^2^)

**Table 2:** Serum concentrations of fasting blood glucose (FBG), lipid markers, and hs- CRP in the Aloe vera and placebo group

p-value (within group)		After 8-week		Baseline		Serum parameters and groups
placebo	Aloe vera	placebo	Aloe vera	placebo	Aloe vera	
0.97	0.07	119.26±11.19	132.74±13.19	123.00±13.73	111.16±7.84	FBG (mg/dl)
0.23	0.11	113.95±15.78	101.71±9.92	103.74±10.17	132.61±21.92	Triglyceride (mg/dl)
0.11	0.01	137.63±7.23	128.16±5.83	146.26±6.77	141.35±5.83	Cholesterol (mg/dl)
0.34	0.02	118.74±6.87	102.54±4.74	119.84±5.30	112.90±4.96	LDL-C (mg/dl)
0.12	0.002	46.16±2.97	44.45±2.44	41.42±2.96	38.32±2.30	HDL-C (mg/dl)
0.15	0.004	31.84±5.60	18.86±2.51	30.83±4.95	29.95±3.96	hs-CRP (mg/l)

**Table 3:** Comparison of fasting blood glucose (FBG) and serum triglyceride, cholesterol, LDL, HDL, and hs- CRP levels between the Aloe vera group and placebo group

Variable	Model	β	Standard error	Standardized coefficient	%95 CI	p-value
FBG	Crude	-22.82	15.36	-0.17	(-53.72-8.08)	0.14
	Adjusted*	-23.34	15.06	-0.18	(-53.66-6.98)	0.13
Triglyceride	Crude	21.86	15.06	0.18	(-8.43-52.15)	0.15
	Adjusted	24.13	14.82	0.20	(-5.71-53.97)	0.11
Cholesterol	Crude	6.04	6.93	0.09	(-7.90-19.99)	0.39
	Adjusted	9.32	6.81	0.10	(-7.39-20.03)	0.36
LDL-c	Crude	12.39	7.08	0.21	(-1.86-26.61)	0.09
	Adjusted	12.82	7.59	0.22	(-2.49-28.12)	0.10
HDL-c	Crude	-0.41	2.98	0.01	(-6.42-5.59)	0.89
	Adjusted	-0.44	3.05	-0.02	(-6.59-5.71)	0.89
hs-CRP	Crude	9.80	3.71	0.25	(2.33-17.27)	0.011
	Adjusted	10.72	3.94	0.27	(2.78-18.66)	0.010

## Discussion

In the current study, 8-weeks of supplementation with Aloe vera was effective in significantly improving serum lipid profiles and hs-CRP in hemodialysis patients. To the best of authors’ knowledge, this was the first study that investigated the effects of Aloe vera supplementation on cardiovascular risk factors in hemodialysis patients. In line with the current study, Choudhary *et al.* reported a significant reduction of fasting blood glucose (FBG) and lipid markers, and blood pressure by Aloe vera supplementation among 90 non-insulin dependent diabetic subjects (Choudhary et al., 2014 ▶). Further, Aloe vera gel (two 300 mg capsules a day for 2 months) can effectively reduce FPG, HbA1c, TC, and LDL levels in type 2 diabetic patients with high lipid profiles (Huseini et al., 2011 ▶). Furthermore, another study was done on 72 subjects with pre-diabetic symptoms and reported a reduction of FPG, HbA1C, and lipid profile (except HDL) after 8-weeks of Aloe vera supplementation (Alinejad-Mofrad et al., 2015 ▶). Inversely, in an animal-based study, oral intake of Aloe vera gel extract for 21 days resulted in a significant reduction of plasma HDL-c levels and increased plasma status of LDL-c and VLDL-c in diabetic rats (Rajasekaran et al., 2006 ▶). Indeed, another study revealed that Aloe vera supplementation has no significant effect on blood glucose levels and lipid profile in diabetes mellitus type 2 patients (Zarrintan et al., 2015 ▶). It can be suggested that Aloe vera has a high amount of Acemannan that can efficiently improve the blood lipid profile through the expression of genes that are responsible for fatty acids metabolism in liver. Also, the compounds in Aloe vera with the help of intestinal bacteria prevent the absorption of sugar and fat compounds from the intestine and improve the lipid profile (Yazdani et al., 2017 ▶).

High consumption of Aleo vera caused nephrotoxicity including renal tubular damage, papillary necrosis, and nephrolithiasis, occurred by high dose of plant (Luyckx et al., 2002 ▶). There is need for further clinical trials to investigate the exact effects of Aloe vera on CVD risk factors and discover the safe dose for this plant in the hemodialysis patients.

In current study, intake of Aloe vera was effective in reduction of serum hs-CRP. Observational studies have shown positive associations between inflammatory mediators and hemodialysis dependency (Kooshki et al., 2019 ▶). The results of current study were in line with the results of Devarai et al. that was conducted on metabolic syndrome patients and showed a reduction of hs-CRP by oral intake of Aloe vera (Devaraj et al., 2013 ▶). Further, the same result was revealed in male rabbits (Dana et al., 2012 ▶). 

Aloe vera, as a traditional herbal medicine contains over 70 biologically active compounds. Many studies have been conducted on the properties of this amazing substance and it can be stated that the intake of Aloe vera has anti-inflammatory and anti-oxidant properties that can increase the body's immunity (Langmead et al., 2004 ▶). The immunity boosting of Aloe vera may be caused thorough inhibition of reactive oxygen metabolites, prostaglandins, and interleukin-8 production (Langmead et al., 2004 ▶). Further, Aloe vera can effectively express ATP sensor P2X7 receptor and attenuate IL-1β cytokine secretion (Budai et al., 2013 ▶). Furthermore, Aloe vera is effective in reduction of tumor necrosis factor alpha and raising interleukin-10, while it had no effect on the cyclooxygenase-2 (COX2) and prostaglandin E2 (PGE2) levels (Keshavarzi et al., 2021 ▶). Also, another study indicated that the carbohydrate fraction of Aloe vera can scavenge free radicals and prevent cell membrane lysis. This process can reduce the inflammatory mediators with an increase in antioxidant enzymes such as superoxide dismutase^ (^SOD) and catalase enzymes (Govindarajan et al., 2021 ▶). 

The limitation of the study was lack of assessment of the effect of Aloe vera supplementation on vascular inflammation markers and apo-lipoproteins. Therefore, further studies in clinical settings should assess these effects and the involved mechanisms.

Our findings justify the role of Aloe vera in reduction of the serum lipid profiles and hs-CRP, as the risk factors of CVD in hemodialysis patients. The study suggests that Aloe vera supplementation might be useful as a complementary therapy along with the usual treatments for the management of cardiovascular complications in hemodialysis patients.

## Conflicts of interest

The authors have declared that there is no conflict of interest. We also declared no

financial or other conflict with Barij Essence Kashan Company.
